# On Pelvic Measurements

**Published:** 1861-02

**Authors:** W. H. Byford

**Affiliations:** Prof. of Obstetrics in the Med. Dep. of Lind University


*On Pelvic Measurements.* By W. H. Byford, M. D., Prof. of Obstetrics in the Med. Dep. of Lind University.—The subject of pelvic measurement is of so much importance, obstetrically, that various modes and instruments have been devised and invented for its correct accomplishment at different times; yet there is no one that gives exact results for all parts of the cavity and straits, and probably all combined, as they are now used, will only enable us to arrive at proximate instead of absolute certainty in this respect. I have examined the writings of all the authors of books and articles in the periodicals within my reach, and the result is as I have stated.

Under these circumstances, I hope it will not be regarded as presumption for me to give a plan which I have taught for the last three years, and demonstrated to the medical classes who have honored me with their attention. These demonstrations have been upon the cadaver entirely, and I have only tried it once upon the parturient patient; but I believe the common sense of the plan will commend itself to the profession sufficiently to insure a trial. The instrument I use is Baudelocque's calipers. The instrument should be constructed with flexible and movable shanks, so that the convexity of their axis can de altered, or that they may be entirely straightened, or one flexed more than the other. The scale for measurement between the shanks cannot be relied upon, in the altered flexures of them, for anything but a rule by which we may readjust the instrument after withdrawing, so that we may measure the distance between the balls upon the extremities, and thus find how far they were separated when in the pelvis. Bearing this in mind, when we wish to measure the antero-posterior diameter, we place the balled extremity of one of the shanks on the upper part of the inner surface of the symphysis pubis, and the other opposite to it upon the external surface, observe the position of the shanks upon the scale, remove the instrument, replace the shanks upon the scale as they were while applied, and then measure the distance between the points. This is the thickness of the pubis and the soft parts covering it, inside and out. Now place the point of one of the shanks upon the promontory of the sacrum, and the other upon the spinous process of the last lumbar vertebra, observe the marks upon the scale, withdraw the instrument, readjust the shanks upon the scale, and measure the distance between the points, when you will have the thickness of the pelvic wall of the superior strait. Now place the point of one shank on the front of the pubis at its upper edge, and the other on the spinous process of the last lumbar vertebra, and this will give us the thickness of both anterior and postericr walls, and the plan of the superior strait; by substracting the two former from this whole measurement, we get the exact measurement of the strait itself. Now by introducing one shank into the vagina, and placing it upon the middle of the linea ilio-pectinea, and placing the other upon the great trochanter outside opposite, we have the thickness of the side wall of the superior strait. Place one point on each great trochanter, and we have the aggregate measurement from one side to the other. By deducting the thickness of the walls, thus ascertained, from the whole sum, we have the exact length of the bilateral line of the superior strait.

It seems to me that there is nothing more simple and exact than the above mode of arriving at correct measurements of the superior strait. The same *mode* of proceeding will give as satisfactory results if applied to the cavity or inferior strait. I hope that members of the profession, who have charge of institutions where they can test the validity of the above mode of measuring the pelvis, will give it a trial, and publish their experiments and conclusions.—*American Journal of Medical Sciences.*

